# Supplementary material for: Feedback and feedforward control are differentially delayed in cerebellar ataxia
Source: bioRxiv. 2025 Feb 10:2025.02.09.637327. Preprint. [Version 1] doi: 10.1101/2025.02.09.637327 (PMC11844357; doi:10.1101/2025.02.09.637327)
Supplement: 1 [file NIHPP2025.02.09.637327v1-supplement-1.pdf]

bioRxiv preprint doi: https://doi.org/10.1101/2025.02.09.637327; this version posted February 10, 2025. The copyright holder for this preprint (which was not certified by peer review) is the author/funder, who has granted bioRxiv a license to display the preprint in perpetuity. It is made available under aCC-BY 4.0 International license.

6 Supplementary materials

6.1 Model Fitting for Feedforward and Feedback Pathway

A

Table III: Model Candidates

**Model Name**	**Transfer Function**
PF Model 1	$ke^{-s\tau}$
PF Model 2	$\frac{k_1}{s+k_2}e^{-s\tau}$
PB Model 1	$\frac{k}{s}e^{-s\tau}$
PB Model 2	$\frac{k_1}{s+k_2}e^{-s\tau}$

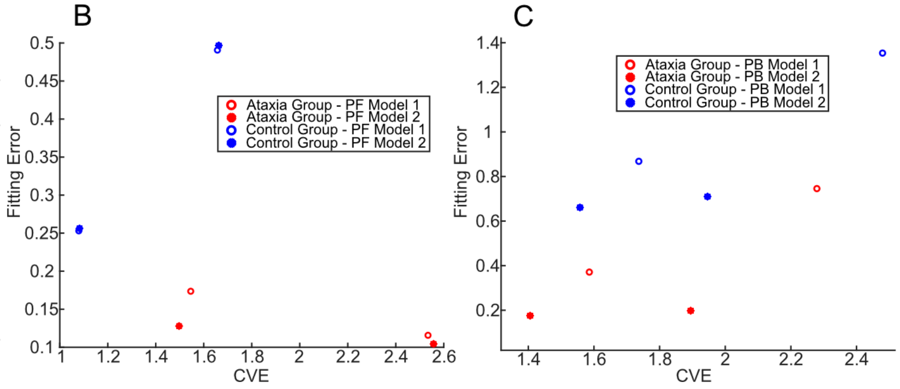

Figure 10: **Model Fitting for Feedforward and Feedback Pathway. A. Top two model candidates for the open loop feedforward pathway (PF) and open loop feedback pathway (PB). B. Model fitting performance for feedforward pathway.** The figure displays the fitting error and cross-validation error (CVE), with two data points for each model candidate corresponding to the x-axis and y-axis fitting. PF model 2 closely resembles PF model 1, but with one additional parameter. Therefore, we select PF model 1, a pure-gain-with-time-delay model, i.e. $ke^{-s\tau}$ as the best-fitted model for the feedforward pathway. Same for the control group and ataxia group. **C. Model fitting performance for feedback pathway.** The PB model 2 exhibits smaller fitting error and smaller cross-validation error (CVE). We select PB model 2, a leaky-integrator-with-time-delay model, i.e. $\frac{k}{s+a}e^{-s\tau}$, as the best-fitted model for the feedback pathway. Same for the control group and the ataxia group.

We listed the top two model candidates for both the feedforward and feedback pathways in Fig. 10A. We have also plotted the model fitting performance, showing the cross-validation error (CVE) versus the fitting error in Fig. 10B,C.

6.2 Preview Filter Modeling

Using the model fitting techniques described in Methods, we created a best fit model of M for both the ataxia group and control group. The same model structure fit both groups, with slightly different parameters (Fig. 11). The model has the following form:

$$M(s) = \overbrace{\frac{a_0}{s+b_0}e^{+s\tau_0}}^{\text{low-pass ``far'' filter}} + \overbrace{\frac{a_1}{s+b_1}e^{-s\tau_1}}^{\text{high-pass ``close'' filter}} \quad (15)$$

The first term is a low-pass filter (LPF) with time lead τ_0, capturing the fact that participants can use "future" information from the preview provided. The second term is a high-pass filter (HPF) with a time delay τ_1, corresponding to accentuating rapid changes of the actual target.

bioRxiv preprint doi: https://doi.org/10.1101/2025.02.09.637327; this version posted February 10, 2025. The copyright holder for this preprint (which was not certified by peer review) is the author/funder, who has granted bioRxiv a license to display the preprint in perpetuity. It is made available under aCC-BY 4.0 International license.

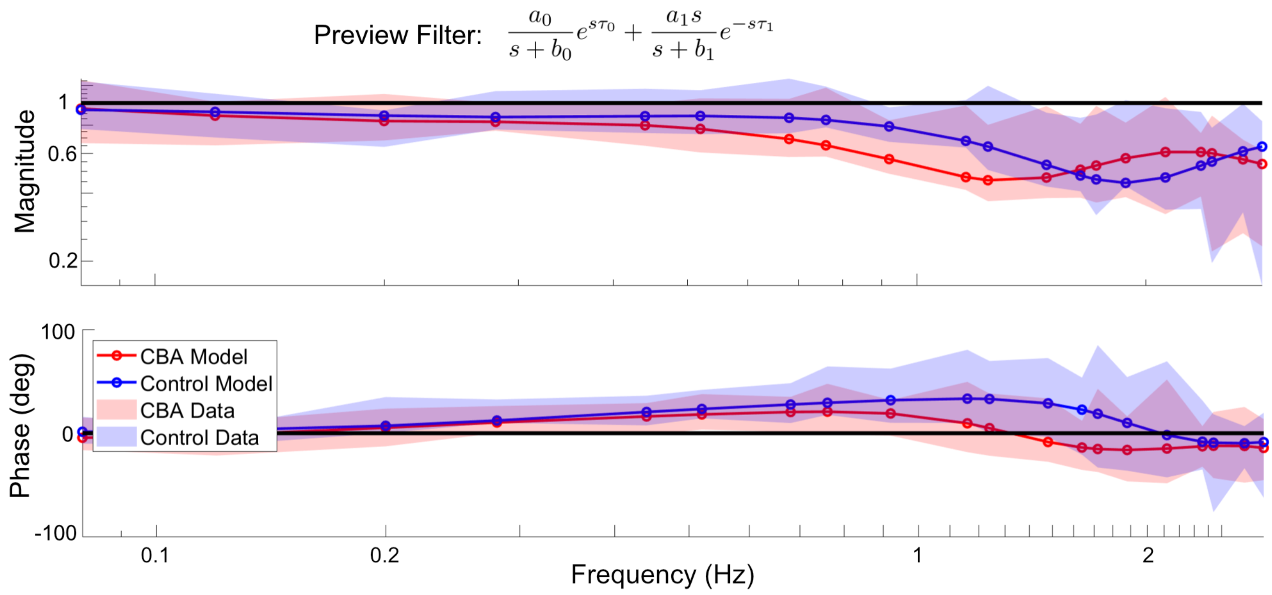

Figure 11: **Model of preview filtering.** The model of the preview filtering, M, introduced in Fig. 6C, fit to the ataxia group's and control group's data. The best-fit model is one that combines a low pass filter with a time lead ($\frac{a_0}{s+b_0}e^{s\tau_0}$) and a high pass filter with a time delay ($\frac{a_1 s}{s+b_1}e^{-s\tau_1}$). The fitted model for control group is $\frac{1.7}{s+1.7}e^{0.4*s} + \frac{0.6*s}{s+2.7}e^{-0.01s}$, and for ataxia group is $\frac{0.8}{s+0.8}e^{0.5*s} + \frac{0.6*s}{s+1.2}e^{-0.02s}$.

bioRxiv preprint doi: https://doi.org/10.1101/2025.02.09.637327; this version posted February 10, 2025. The copyright holder for this preprint (which was not certified by peer review) is the author/funder, who has granted bioRxiv a license to display the preprint in perpetuity. It is made available under aCC-BY 4.0 International license.

The structure of the model suggests what pieces of information subjects are using from the reference preview. There are two important components to the model: a time-lead component based on a "future/far point" and a time-lag component based on a "near point". The time-lead component of the reference filtering is a low pass filter. This suggests that for future points in the preview trajectory, subjects may focus on the low frequency movements, meaning the general motion path of the future points is of interest, not precisely capturing the more rapidly oscillating components of the movement. Conversely, the time-lag component of the reference filtering is a high pass filter. This suggests that for points on the trajectory closer to the reference, there is a focus on precisely capturing the high frequency, seemingly erratic, parts of the movement trajectory. These two components in tandem suggest that subjects are using information from multiple time-points along the reference preview to improve their tracking performance. Note model is phenomenological and highly simplified (only two terms), but is consistent with participants using multiple points along the preview trajectory.
